# Exploring Environmental Element Monitoring Data Using Chemometric Techniques: A Practical Case Study from the Tremiti Islands (Italy)

**DOI:** 10.3390/molecules31020232

**Published:** 2026-01-09

**Authors:** Raffaele Emanuele Russo, Martina Fattobene, Silvia Zamponi, Paolo Conti, Ana Herrero, Mario Berrettoni

**Affiliations:** 1 Chemistry Division, School of Science and Technology, University of Camerino, Via Madonna delle Carceri-ChIP, 62032 Camerino, MC, Italymartina.fattobene@unicam.it (M.F.);; 2 Department of Chemistry, Faculty of Sciences, Universidad de Burgos, Plaza Misael Bañuelos s/n, 09001 Burgos, Spain

**Keywords:** environmental monitoring, elements, BVOCs, *Posidonia oceanica*, sediments, chemometrics, N-Way techniques, PARAFAC

## Abstract

Environmental element monitoring is essential for assessing environmental quality, identifying pollution sources, evaluating ecological risks, and understanding long-term contamination trends. Modern monitoring campaigns routinely generate large volumes of complex data that require advanced analytical strategies. This study applied chemometric techniques to analyze elements and BVOCs (biogenic volatile organic compounds) measured from *Posidonia oceanica* and related environmental matrices (seawater, sediment, and rhizomes) during three sampling campaigns in the Tremiti Islands (Italy). Twenty-two trace elements were quantified, and BVOC profiles were obtained from the leaf samples. The dataset was analyzed using a combination of univariate visualizations, unsupervised and supervised multivariate techniques, and multi-way methods. PCA (Principal Component Analysis) and PLS-DA (Partial Least Squares-Discriminant Analysis) revealed distinct spatial (leaf section) and temporal (sampling period) trends, supported by consistent elemental markers. A low-level data fusion approach integrating BVOC and element data improved group discrimination and interpretability. PARAFAC (PARAllel FACtor analysis) applied to a three-way array successfully separated background trends from meaningful compositional changes, uncovering latent structures across chemical, spatial, and temporal dimensions. This work illustrates the usefulness of chemometrics in environmental monitoring and the effectiveness of combining multivariate tools and data fusion to improve the interpretability of complex environmental datasets. The methodology used in this study is fully generalizable and applicable to other environmental multi-way datasets.

## 1. Introduction

Environmental monitoring is a tool for assessing environmental conditions and trends, supporting policy development and implementation, and providing information for reporting to national policymakers, international forums, and the public [1,2]. Human activities and natural events can significantly affect the balance of ecosystems, leading to environmental problems such as pollution, biodiversity loss, and climate change [3,4,5,6]. Environmental monitoring has become a critical component of environmental risk assessment and remediation strategies. Among various environmental issues, the presence of trace metals and metalloids in water, soil, and air is of particular concern because of their toxicity, persistence, and potential to accumulate in living organisms [7,8]. Thus, unlike many organic and radionuclide pollutants, metals do not decay with time, cannot be degraded, and often become more concentrated as they move through environmental and biological systems [9].

Environmental element monitoring involves the systematic collection and analysis of environmental matrices, such as water, soil, sediment, and biota, to detect and quantify the presence of contaminants [2,10]. For elements (metals, metalloids…), the main goals are to (i) establish background levels, (ii) detect anomalies or hotspots of pollution, (iii) identify sources of contamination (natural vs. anthropogenic), and (iv) assess temporal and spatial trends. Traditional univariate approaches are still widely used because they are simple and easy to interpret, but they often fall short in handling large, complex datasets, especially when several elements and environmental variables/indexes are involved across different geographical locations or timelines [11]. Thus, it becomes difficult to understand the complex relationships that could exist between variables and to identify pollution sources or trends over time. The integration of chemometric techniques has emerged as a powerful tool that allows researchers to visualize and identify patterns, classify samples, and gain insights from multivariate data collected during environmental monitoring campaigns involving element contamination [12,13,14,15].

By applying chemometrics to real-world monitoring data, scientists can uncover hidden patterns, differentiate between natural and anthropogenic element contributions, and develop more informed risk assessments [16,17,18]. Therefore, it is desirable to adopt more advanced tools than traditional ones, starting from advanced univariate techniques and moving towards more powerful chemometric methods. For example, Box-and-Whisker plots and row profile charts offer clearer insights into data variability and visualization than other methods do [19]. Exploratory multivariate techniques, such as Principal Component Analysis (PCA), PARAllel FACtor (PARAFAC) analysis, and Multivariate Curve Resolution-Alternating Least Squares (MCR-ALS), allow for the identification of patterns and latent structures in two-, three-, or higher-way datasets [20,21,22,23]. Furthermore, classification tools such as Partial Least Squares-Discriminant Analysis (PLS-DA) are effective in showing pollution sources between groups [19]. Another chemometric tool widely employed in chemistry, biology, and biomedicine is ANOVA Simultaneous Component Analysis (ASCA) [24]. This is a multivariate extension of ANOVA that treats multiple responses by combining the variance factorization and inference capabilities of ANOVA with the exploratory power of PCA [25]. In simpler terms, the spatial and temporal factors are organized in an experimental design matrix, and the measured analytes are treated as multiple responses. This approach helps us understand whether these factors have a significant influence on data structure.

In the literature, very few studies have explored the use of multi-way techniques, such as PARAFAC, to analyze data from environmental monitoring, despite the multidimensional nature of the datasets. For example, some authors have applied PARAFAC to investigate the mobility of elements in sediments using sequential or single-step extraction protocols [26,27] or to analyze the distribution of elements in fish, sediments, and river water [28]. However, these contributions remain scarce, and the use of multi-way methods is still far from being a common practice in environmental chemistry. This underscores the relevance of the present study, whose objective is not only to demonstrate the advantages of these tools in the analysis of real-world environmental data but also to promote their broader adoption within this scientific community.

It is important to underline that the success of chemometric tools depends largely on the quality and design of the sampling process. Sampling is not a secondary step but a critical component of the analytical workflow. To extract meaningful patterns and reliable evidence of variability from environmental data, it is essential to carefully plan the sampling strategy, ensuring sufficient variability across the dimensions of interest (e.g., spatial, temporal, or environmental conditions) [2]. Without this, even the most sophisticated multivariate tools may fail to provide useful information. In other words, chemometric methods are not meant to create information where no information exists; they are designed to reveal existing structures that must be captured during the data acquisition.

Thus, this paper presents a practical case study, such as a guide, in which multivariate techniques are essential for the in-depth analysis of environmental datasets with elements and biogenic volatile organic compound (BVOC) concentrations. We explored how data preprocessing, multivariate or multi-way analysis (such as PCA and PARAFAC), and classification models (such as PLS-DA) can reveal important and hidden contamination patterns, support pollution source identification, and support sustainable management of ecosystems, thus improving decision-making in environmental monitoring programs.

## 2. Results and Discussion

### 2.1. Data Visualization

The first essential step in any chemical analysis is the comprehensive visualization of the dataset. This preliminary phase allows researchers to gain a general understanding of the data structure, detect outliers, and identify potential trends or groupings in the data. In the field of environmental monitoring, where datasets are often complex and multidimensional, effective data visualization is critical to ensure meaningful interpretation and guide subsequent preprocessing and modelling steps. Among the most commonly used univariate exploratory tools in the chemometric community are Box-and-Whisker plots and row profiles [19]. These graphical methods provide complementary insights into the data distribution. The first is particularly useful for evaluating the dispersion and symmetry of the individual variables. For each element or variable, the box lines correspond to lower, median, and upper quartile values. The whiskers extend to the most extreme data points that are not considered outliers, whereas points beyond the whiskers indicate possible outliers that can be investigated further. Row profiles, on the other hand, visualize the variation of all variables across individual samples, offering a sample-wise overview of the data matrix. This technique is particularly informative for detecting abnormal samples or trends over time or space that are relevant to environmental monitoring. Thus, data visualization helps reveal variable-specific patterns, such as skewness, heteroscedasticity, and outlying observations, and supports the selection of suitable scaling methods and preprocessing procedures, such as normalization or transformation, to enhance model performance and interpretability.

**Figure 1:**
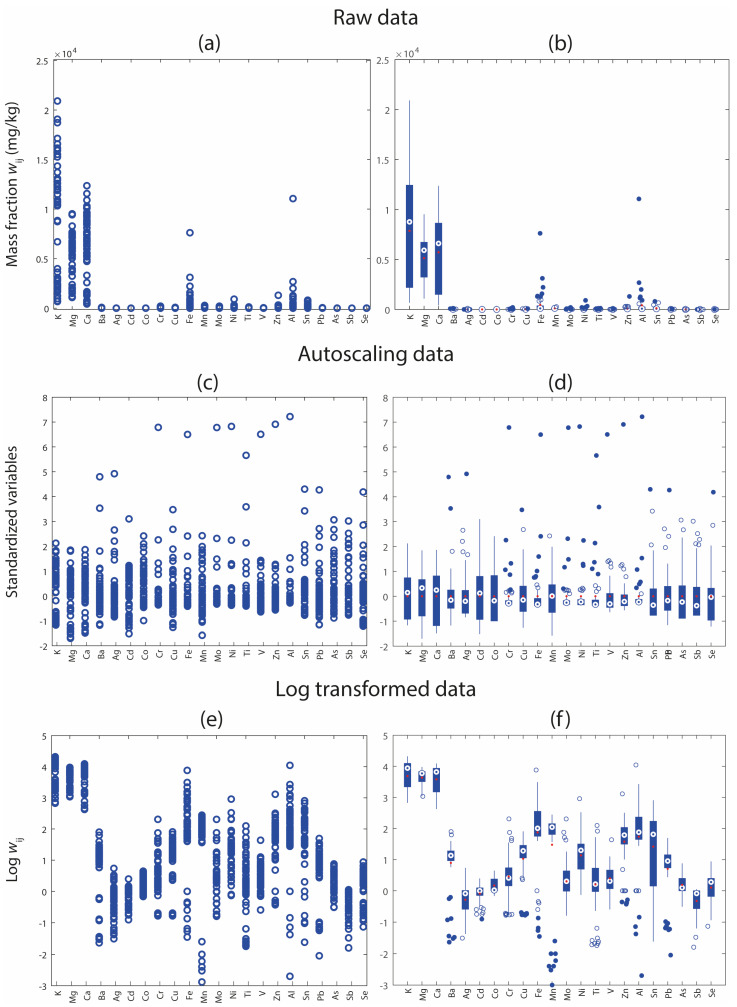
Visualization of elemental mass fraction data under different preprocessing conditions. Row profiles (left column) and Box-and-Whisker plots (right column) are shown for (**a**,**b**) raw data, (**c**,**d**) autoscaled data, and (**e**,**f**) log-transformed data. The interquartile range is shown as a blue box. The robust data range is indicated by vertical blue whiskers. The median is represented by an open blue circle, while the mean is shown as a filled red circle. Outliers are displayed as open circles, whereas extreme outliers are indicated by filled blue circles.

Figure 1 illustrates the effect of different preprocessing strategies on the multivariate dataset containing elemental mass fractions using two different visualization tools: row profile plots (left column) and Box-and-Whisker plots (right column) for raw, autoscaled, and log-transformed data. These preprocessing approaches were selected because they are among the most effective in environmental studies, particularly where the element mass fraction is based on several orders of magnitude. The sequence of displayed elements in the plots follows the order of groups in the periodic table as follows: alkali and alkaline earth metals, transition metals (in alphabetical order), post-transition metals (Al, Sn, Pb), metalloids (As, Sb), and non-metals (Se). Figure 1a shows the row profiles of the raw data. The dominance of a few high-abundance elements (e.g., Ca, Mg, and K) masks the contributions of lower-abundance elements, limiting the ability to detect their variation across samples. To visualize all elements, it is generally preferable to use a broken vertical scale, or alternatively, to adopt a dual-axis scale (for example, one on the left for higher values and one on the right for lower values) as already done in Martina Fattobene et al. [29]. However, this solution was not adopted here, as subsequent data transformations (autoscaling and logarithmic transformation) were applied specifically to mitigate this scaling issue and to clearly highlight the differences between raw and preprocessed data. These transformations make low-abundance elements more visible and allow for a more balanced and interpretable comparison across the full variable set. Figure 1b presents the corresponding Box-and-Whisker plots for the raw data, highlighting the wide range of mass fractions and the presence of numerous outliers for several variables, indicating strong heterogeneity in the dataset. On the other hand, Figure 1c displays the row profiles after autoscaling. This transformation makes all variables on a comparable scale, making patterns and inter-sample differences more easily detectable across the full variable set. Figure 1d shows Box-and-Whisker plots of the autoscaled data, where the interquartile ranges are standardized, and the distribution of values appears more uniform. Finally, Figure 1e illustrates the row profiles of the log-transformed data. Log transformation, which is widely used with environmental data, reduces data skewness and compresses the dynamic range, thereby enhancing the interpretability of both low- and high-abundance elements. Finally, Figure 1f presents the Box-and-Whisker plots of the log-transformed data, revealing a more balanced spread of values across variables, with reduced influence from extreme values and more normally distributed variables.

**Figure 2:**
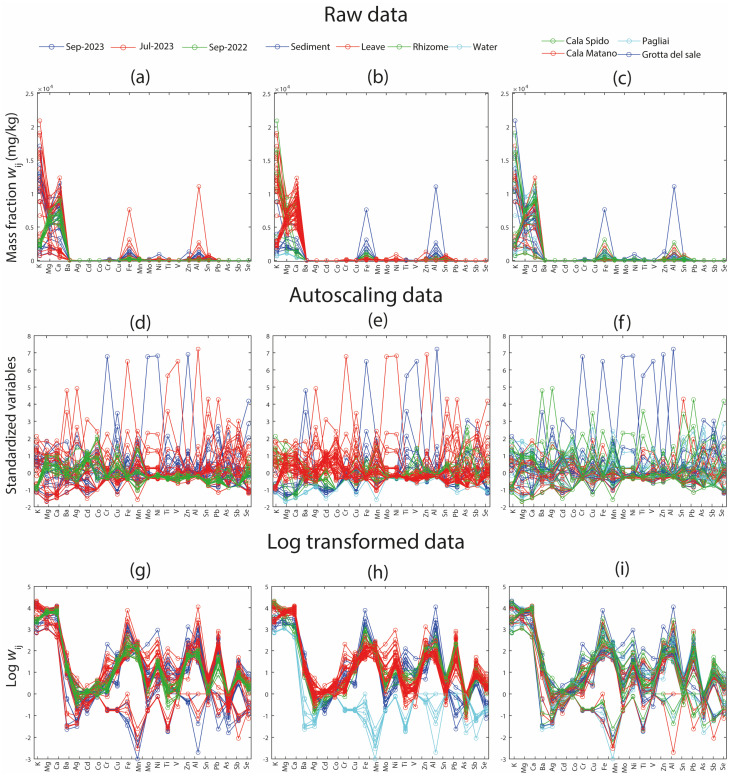
Row profile plots of elemental mass fraction data (in mg/kg) under different preprocessing conditions: raw data (**a**–**c**), autoscaled data (**d**–**f**), and log-transformed data (**g**–**i**). Samples are grouped and color-coded by sampling time ((**a**,**d**,**g**): blue = September 2023, red = July 2023, green = September 2022), environmental matrix ((**b**,**e**,**h**): blue = sediment, red = leaf, green = rhizome, cyan = water), and sampling site ((**c**,**f**,**i**): green = Cala Spido, red = Cala Matano, cyan = Pagliai, blue = Grotta del Sale).

To further explore the structure of the dataset, row profile plots were generated using three grouping criteria: sampling time, environmental matrix, and sampling site (Figure 2). Color coding plays a crucial role in these visualizations as it introduces an additional layer of information, enabling the simultaneous evaluation of chemical variability along with temporal, spatial, or matrix-related patterns. This represents a preliminary step in identifying the factors that contribute to variability and consequently carry relevant information within the dataset.

In the row profiles of the raw data grouped by sampling time (Figure 2a), the September 2022 samples display a markedly different profile than those collected in July and September 2023. This divergence is less apparent when grouping by environmental matrix and sampling site (Figure 2b,c), suggesting that temporal variability may play a key role in the observed differences. However, it is important to note that no definitive conclusions can be drawn from raw data alone, as differences in scale and magnitude between variables make the profiles non-comparable without appropriate preprocessing.

In the autoscaled data (Figure 2d,e), a single sample stands out due to a distinct trend characterized by notably higher mass fraction of elements such as Cr, Mo, and Ni. This deviation suggests the presence of a potential outlier. As discussed in the following section, this specific sample was identified as an outlier using PCA diagnostic tools (Hotelling’s T^2^ and Q-residuals).

Finally, in the log-transformed data (Figure 2g–i), a distinct subset of the samples appears to be clearly separated from the rest (Figure 2h). This separation reflects environmental differences and highlights the usefulness of log transformation in enhancing the detection of latent patterns in datasets with a high dynamic range.

Overall, these visual tools offer fundamental first insights into the structure and quality of environmental datasets, allowing researchers to identify anomalies, guide preprocessing choices, and select variables or sample groups for more in-depth investigation.

### 2.2. Unsupervised Approach: PCA

To extract meaningful information from complex datasets, it is essential to maximize the amount of variability captured. PCA is one of the most widely used exploratory techniques for this purpose because it reduces the dimensionality of multivariate data while preserving the most significant sources of variance [30].

Before interpreting the PCA results, potential outliers were identified using two diagnostic indices, Hotelling’s T^2^ and Q-residuals on PCA scores. These metrics help identify samples that deviate significantly from the overall multivariate structure. It is important to note that both the Q and T^2^ indices depend on the number of principal components retained in the model [31]. Additionally, the K-Nearest Neighbors (KNN) score distance (with k = 3) was employed as an alternative method specifically suited for detecting distance-based outliers [32]. It gives the average distance to the nearest neighbors in score space for each sample, and it is an indication of how well sampled the given region of the score space was in the original model. Outlier diagnostics were conducted using four principal components, as described below.

**Figure 3:**
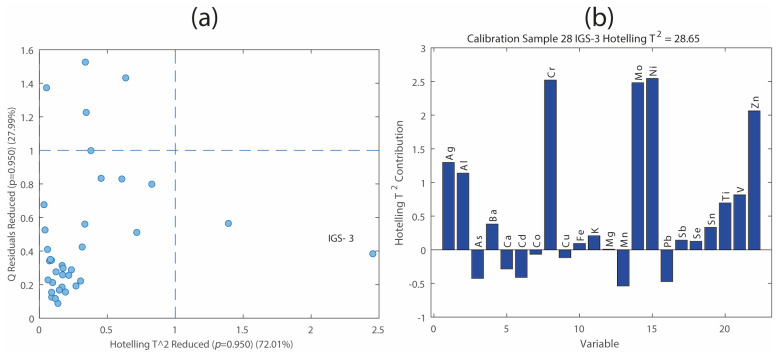
(**a**) Hotelling’s T^2^ vs. Q-residuals plot for the PCA model built on the leaf dataset. Dashed lines represent the 95% confidence limits for the T^2^ and Q indices. (**b**) Variable-wise contribution to Hotelling’s T^2^ index for sample IGS-3. Blue dots represent the samples.

**Figure 4:**
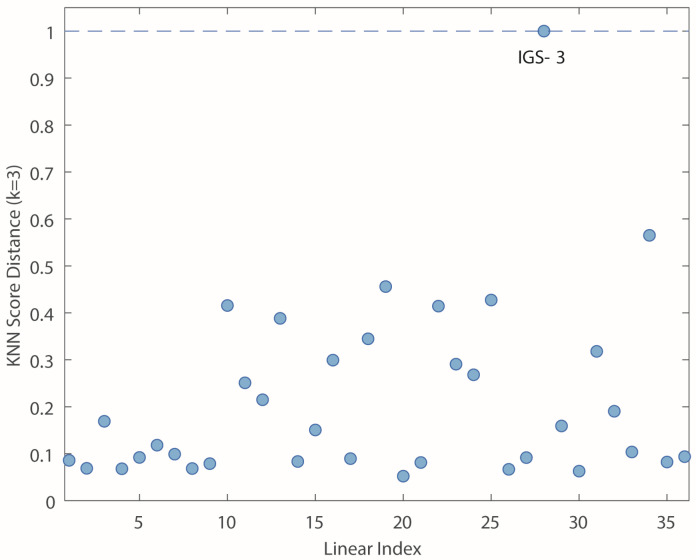
K-Nearest Neighbors (KNN) score distance plot (k = 3) for data based on the leaf dataset. Blue dots represent the samples [29].

Figure 3a shows the Q-residuals versus Hotelling’s T^2^ plot for the leaf matrix. Typically, outliers are samples located in the upper-right quadrant of the plot, that is, samples exhibiting both high Q and Hotelling’s T^2^ values above the established threshold values (usually, the probability level is 95%). However, analytical decisions should not rely solely on this general rule because each dataset has specific characteristics. In this case, the sample IGS-3 shows a particularly high Hotelling’s T^2^ value, indicating that it is far from the center of the PCA model in the score space. Figure 3b displays the variable contributions to Hotelling’s T^2^ index for the sample IGS-3. The most influential elements driving the outlier status were Cr, Mo, Ni, and Zn, which appeared in abnormally high mass fractions in this sample. As further confirmation, the KNN score distance analysis (Figure 4) also detected IGS-3 as a clear outlier, reinforcing the evidence from Hotelling’s T^2^ and Q-residual analyses.

Together, these outlier detection tools provide complementary perspectives, ensuring a suitable evaluation of data quality and improving the reliability of subsequent multivariate analyses. An alternative strategy for handling atypical samples is the application of robust PCA, which is specifically designed to reduce the influence of outliers during model construction by employing robust estimators of the location and scale [33,34].

**Figure 5:**
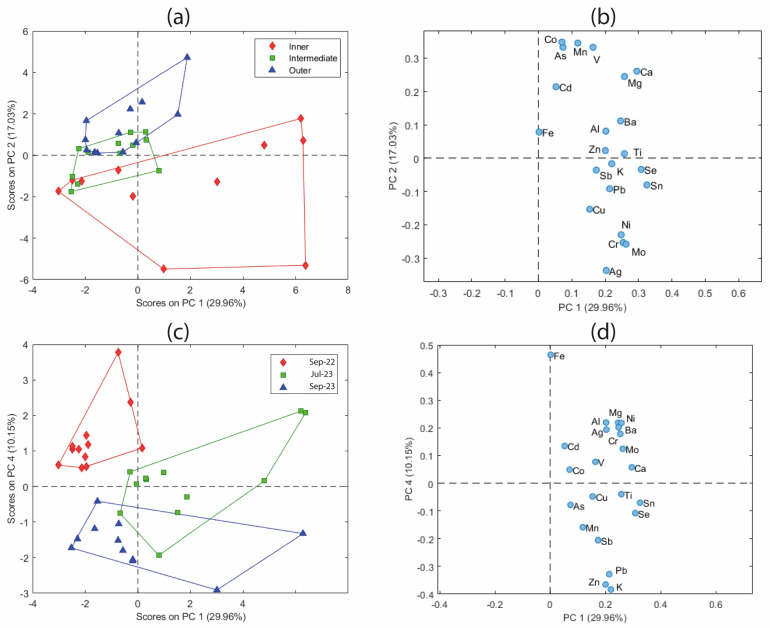
PCA score and loading plots: (**a**,**b**) Grouping by leaf section. (**c**,**d**) Grouping by sampling campaign.

After identifying and excluding outliers (IGS-3), PCA was conducted once again, after autoscaling, using the remaining leaf samples to investigate their underlying multivariate structure. Table 1 shows the eigenvalues and explained variance associated with the first seven principal components retained from the PCA model applied to the elemental dataset of leaf samples. Four components were retained in the final model, as they accounted for 73.20% of the total variance and captured the major sources of variability in the dataset. PC3 was not included in the interpretation, as it did not provide meaningful insights into the underlying data structure or contribute to relevant sample grouping patterns. The score plots based on PC1 and PC2 (Figure 5a,c), which together account for 46.99% of the total variance, provide insights into the differentiation among leaf sections and the influence of sampling periods, whereas the corresponding loading plots (Figure 5b,d) highlight the elemental contributions responsible for the observed variability. Thus, (i) PC1 expresses the overall elemental contents; (ii) PC2 expresses the contrast between leaf sections (contrast mainly between Co, As, V, Ca, Mg, on the one hand, and Al, Mo, Cr, Ni, on the other); (iii) PC4 interprets the differences between sampling periods (contrast mainly between elements such as Fe, Mg, Al, Ni, and the elements K, Zn, Pb).

In Figure 5a, the samples are color-coded according to the leaf section (inner, intermediate, and outer), and a separation is observed along PC2. Notably, the intermediate leaves are positioned between the inner and outer samples, as expected, often overlapping with both groups. It is important to note that the classification of intermediate leaves is more susceptible to misclassification than that of the inner or outer sections. This is due to the morphological structure of *Posidonia oceanica*, where the visual distinction between intermediate and adjacent leaf zones is less clear. As a result, some misclassifications can occur during sampling, especially for intermediate leaves, which could affect their positioning in the PCA space. Figure 5b shows that elements such as Co, Mn, As, V, Mg, and Ca contribute positively to PC2 and are therefore closely related to the outer part of the leaves. In contrast, other elements, such as Ag, Mo, Cr, and Ni, contribute negatively to that PC and are therefore more prevalent in the inner leaves. These elements appear to be the key drivers of compositional differences among leaf sections. In other words, this means that the outer section has high values of mainly Co, Mn, As, V, Ca, Mg, etc., and low values of mainly Ag, Cr, Ni, etc., while the opposite situation occurs for the inner section, and the intermediate section has intermediate values in every case. To explore temporal trends, a second score plot (Figure 5c) was constructed using PC1 and PC4, the latter explaining 10.15% of the variance. The samples are color-coded according to the sampling period. A clear separation is observed, particularly for the September 2022 samples, which form a distinct cluster. This suggests potential seasonal influences or occasional environmental events that affect the elemental composition. The corresponding loading plot (Figure 5d) shows that PC4 is positively influenced by elements such as Fe, Mg, Al, and Ni, among others, and negatively influenced by Pb, Zn, and K. In other words, this means that the Sep-22 sampling presents high values mainly of Fe, Mg, Ni, etc., and low values mainly of K, Pb, and Zn, whereas the opposite situation occurs for Sep-23, and for the Jul-23 sampling, intermediate values are observed for both the first and the second group.

To further demonstrate the utility of PCA, a second analysis was performed after autoscaling on a fused dataset obtained from the September 2022 sampling campaign, which included both trace element mass fractions and BVOC emission profiles from leaf samples grouped by organic class (Section 3.1). As in the above analysis, outliers were first evaluated using Hotelling’s T^2^ versus Q-residuals, considering two components. No outliers were revealed.

**Figure 6:**
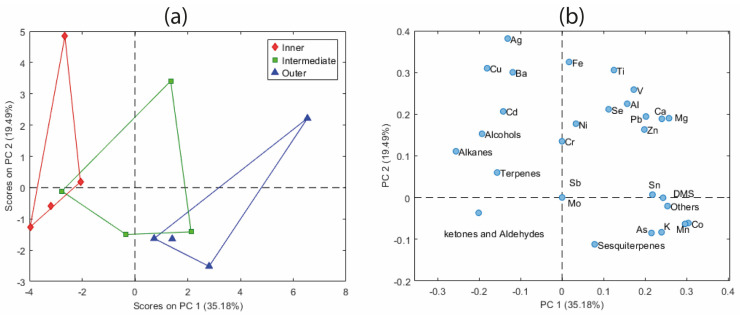
PCA applied to the fused dataset integrating trace element mass fractions and BVOC emission profiles from leaf samples: (**a**) score plot of PC1 vs. PC2; (**b**) corresponding loading plot.

Figure 6a shows the score plot based on PC1 and PC2 for this new dataset, which explained 35.18% and 19.49% of the variance, respectively, after autoscaling. A trend similar to the previous PCA result is observed when looking at PC1 instead of PC2: intermediate leaves are positioned between the inner and outer leaf sections, showing a clearer separation than previously. This consistent PC spatial configuration reinforces the pattern of chemical differentiation along the leaf gradients. The corresponding loading plot (Figure 6b) shows the variables driving the observed separation. Specifically, the outer leaf samples are characterized by the following features:

- High values of variables located in the more positive part of PC1, including Co, Mn, or Mg, Ca, K (which confirmed the results of the above analysis), in association with dimethyl sulfide (DMS), the variable “others”, and sesquiterpenes.
- Low values of alkanes, ketones, and aldehydes, alcohols, and terpenes, with associated Ag, Cu, Ba, and Cd.

The exact opposite (low values for the first group and high for the second one) occurs for the internal section, while the intermediate section presents intermediate values in every case for all the variables considered, which contributed significantly to the observed group separation. Thus, the contrast between the first group of variables and those of the second group is the basis of the separation of the leaf samples into the internal, intermediate, and external sections and evidence of group structure in the PCA space. The ratio of dimethylsulfoniopropionate (DMSP) to dimethyl sulfoxide (DMSO) has been demonstrated to serve as an indicator of oxidative stress and has been correlated with element accumulation [35]. Dimethyl sulfide, a common precursor of both compounds, plays a central role in this redox-related pathway.

This approach demonstrates that integrating different analytical techniques within a single dataset through low-level data fusion can yield complementary information, thereby enhancing the interpretative power of multivariate analysis. Most importantly, it provides a more comprehensive understanding of complex environmental systems, making it particularly valuable for environmental monitoring applications in which multiple sources of chemical variability must be assessed simultaneously.

### 2.3. Supervised Approach: PLS-DA

PLS-DA can be considered a supervised version of PCA that combines dimensionality reduction with group discrimination. While PCA identifies the directions of maximum variance in the dataset without considering sample classes, PLS-DA incorporates class labels during model construction, allowing for improved separation between predefined groups [19].

**Figure 7:**
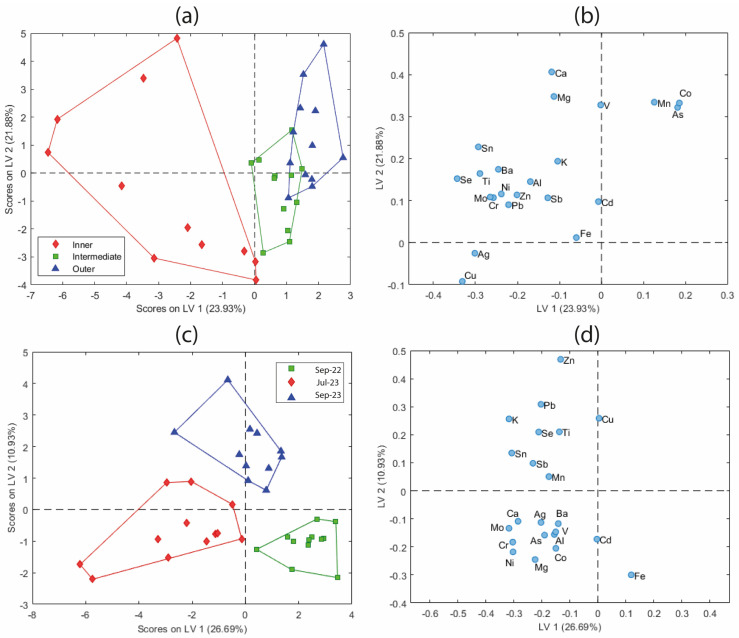
PLS-DA score and loading plots: (**a**,**b**) Grouping by leaf section. (**c**,**d**) Grouping by sampling campaign.

In this study, PLS-DA was applied to the leaf dataset to explore the discrimination between groups based on both leaf section and sampling period. Figure 7a shows the score plot based on the first two latent variables (LV1 and LV2) selected for the leaf model, which together explain 45.81% of the total variance in X. Samples are color-coded according to the leaf section: inner, intermediate, and outer. A clear separation is observed, particularly between the inner and outer samples along LV1, whereas the intermediate samples occupy an intermediate position with some overlap, consistent with the PCA results. This pattern reinforces the hypothesis that classification uncertainty is greater for intermediate leaves due to the morphological complexity of *Posidonia oceanica*, although the supervised approach using PLS-DA differentiates them to a greater extent.

The corresponding loading plot (Figure 7b) shows that inner leaves are associated, for example, with higher levels of elements such as Cu, Ag, or Sn (those with the most negative loadings in LV1), while outer leaves are linked to higher mass fractions of Mn, As, and Co (positive LV1). Elements positioned on opposite sides of LV1 are negatively correlated, indicating that outer leaves tend to have lower mass fractions of Cu, Ag, and Mo.

To assess temporal trends, a second PLS-DA model was constructed using the sampling period as the classification criterion. The score plot (Figure 7c) based on LV1 and LV2 (explaining 37.62% of the variance in X) reveals a clear temporal separation, which is much more defined than that obtained using PCA (Figure 5c). It is observed that this increased differentiation now depends on both latent variables. In particular, the first (LV1) separates the period Sep-22 from Jul-23, while the second (LV2) separates the period Sep-22 from the other two. The corresponding loading plot (Figure 7d) highlights the elemental contributions that drive this good temporal differentiation. Overall, samples from September 2023 were clearly associated with higher levels of Zn, Pb, and Cu, and those from July 2023 were associated with Mg, Ni, or Cr, among others, whereas September 2022 samples were characterized by high mass fractions of Fe and Cd.

Therefore, PLS-DA can be particularly useful in environmental monitoring studies, where high data variability can hide meaningful trends in unsupervised methods such as PCA. As PLS-DA is a supervised technique, it incorporates prior knowledge of sample groupings, allowing the model to focus on the variations that are relevant for discrimination between predefined classes. Thus, it is possible to highlight patterns that may remain hidden using an exploratory approach. In the present case, although PCA suggested some degree of separation between groups, PLS-DA achieved a clearer and more structured distinction, confirming the same underlying information while enhancing interpretability. This demonstrates the added value of supervised methods, such as PLS-DA, when the analytical objective is to reveal and study class-specific patterns within complex multivariate datasets with high variability.

### 2.4. N-Way Unsupervised Approach: PARAFAC

**Figure 8:**
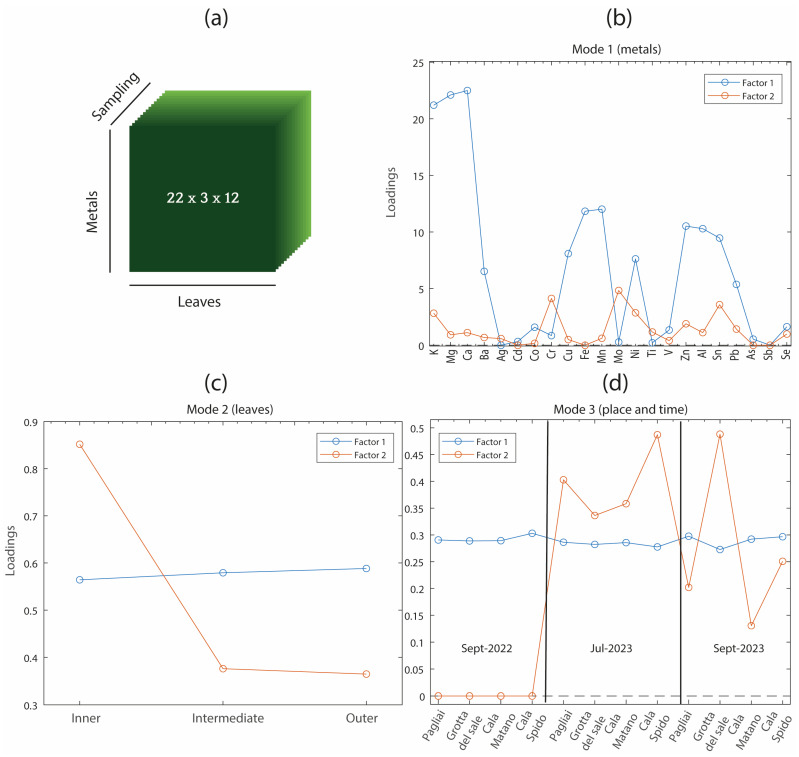
PARAFAC model results: (**a**) Structure of the three-way data array (elements × leaf sections × sampling units). (**b**) Loadings for mode 1 (elements). (**c**) Loadings for mode 2 (leaf sections). (**d**) Loadings for mode 3 (sampling site and time).

To further explore the structure of the dataset and reveal the underlying sources of variability across multiple dimensions, the data were arranged in a data cube and analyzed using PARAFAC [23]. PARAFAC is a multi-way extension of PCA that can decompose three-way data arrays. In this case, a data cube of dimension 22 × 3 × 12 was obtained; the first dimension refers to the element composition way (mode 1), the second one to the leaf section (mode 2), and the third one to the sampling places and temporal way (mode 3). Figure 8a shows the structure of the three-way data array, which consists of 22 elements × 3 leaf sections × 12 spatio-temporal sampling units (4 places × 3 months). Unlike traditional PCA, which requires a two-dimensional matrix, PARAFAC preserves the original multi-way structure, allowing for a more interpretable and holistic representation of the interactions among variables, samples, and conditions. A two-factor model was estimated from the data cube, which explained 98.58% of the variance; a consistency diagnostic index (CORCONDIA) of 100% guaranteed the trilinearity of the data [36].

The loading profiles for each mode are shown in Figure 7b–d. In mode 1 (elements, Figure 8b), Factor 1 was associated with high contributions from elements such as Ca, K, and Mg, whereas Factor 2 was driven by elements such as Ni, Cr, and Mo. These distinct patterns suggest different geochemical or physiological behaviors of the element groups. Mode 2 (leaf sections, Figure 8c) shows that Factor 1 remains practically constant across all sections, whereas Factor 2 strongly differentiates the inner leaves from the intermediate and outer leaves. Mode 3 (sampling site and period, Figure 8d) reveals the different temporal and spatial evolutions of the two factors. While Factor 1 remains constant across all sites and times, Factor 2 increases notably in July and September 2023 and appears to vary more across locations. This suggests that the data cube information captured by Factor 2 may be associated with episodic environmental conditions or anthropogenic influences that affect specific sites during the campaign.

It is important to underline that PARAFAC was able to distinguish the overall average element content (captured by Factor 1) from the variation patterns that truly describe the samples (captured by Factor 2). Thus, it can be concluded that the inner parts of the leaves collected during the 2023 campaigns contained higher amounts of elements such as Cr, Mo, Ni, and Sn than those collected in 2022. Notably, none of the previously applied techniques (PCA or PLS-DA) could provide this type of information, highlighting the added value of the multi-way approach in revealing latent trends across all three experimental dimensions.

Although this can be considered a relatively simple case, the potential of PARAFAC becomes even more evident when considering more complex datasets, such as long-term environmental monitoring campaigns conducted across multiple seasons, locations, and years. Unlike PCA or PLS-DA, which use two-dimensional predictor variable tables (*n* × *p*) in which all sources of variability are merged, PARAFAC maintains the natural structure of the data with all its dimensions. In other words, PARAFAC works directly with data cubes or higher-dimensional arrays without the need to unfold them into two-dimensional arrays.

This is very important when the dataset has different sources of variation, such as chemical, spatial, and temporal differences, because PARAFAC can study the full structure of the data and better explore the relationships between variables. Thus, in these situations, using multi-way methods, such as PARAFAC, can be really useful because they help us retain all the information and understand the data better. With other techniques, such as MCR-ALS, which work with unfolded data, it is possible to adopt strategies that also allow the multidimensional structure of arrays of three or higher dimensions to be studied [21]. This highlights its strong potential as an advanced analytical tool for environmental monitoring and assessment across multiple data dimensions.

## 3. Materials and Methods

### 3.1. Dataset

**Figure 9:**
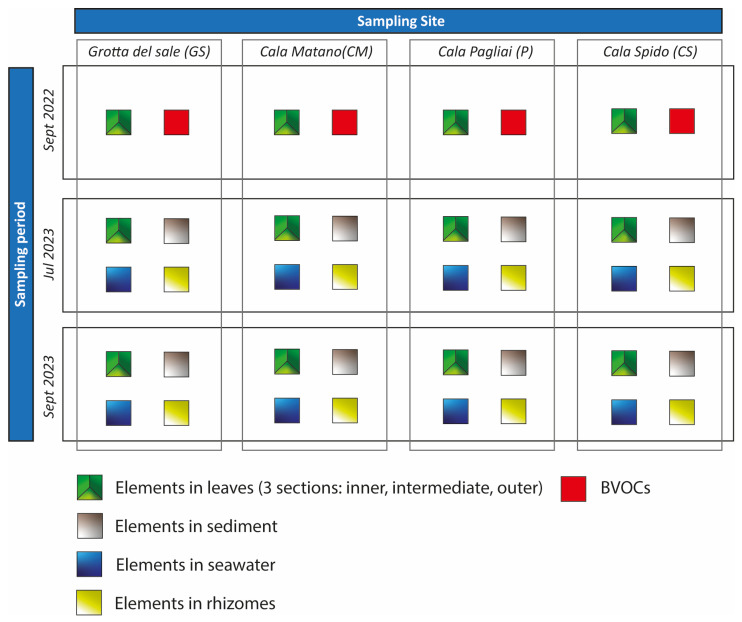
Analysis and monitoring of *Posidonia oceanica* in the Tremiti Islands archipelago.

The dataset analyzed in this study originated from an environmental monitoring campaign focused on *Posidonia oceanica*, a plant species in the Mediterranean ecosystem [37]. Samples were collected from four distinct locations around the Tremiti Islands at three time points (Figure 9). This study involved four environmental matrices: marine sediments, rhizomes, leaves, and seawater. A total of 22 trace elements (Ag, Al, As, Ba, Ca, Cd, Co, Cr, Cu, Fe, K, Mg, Mn, Mo, Ni, Pb, Sb, Se, Sn, Ti, V, and Zn) were quantified using Inductively Coupled Plasma–Optical Emission Spectrometry (ICP-OES) in each collected sample, with results expressed in mg/kg (dry weight). Leaf samples from the first sampling campaign (September 2022) were also analyzed for BVOCs. Their profiles were obtained using HS-SPME/GC-MS and expressed as relative percentages normalized to 100% for each sample. BVOCs were further grouped into major chemical classes: terpenes, sesquiterpenes, alkanes, ketones and aldehydes, alcohols, and other minor compounds. Dimethyl sulfide (DMS) was not included in any class and considered as well because it plays a crucial role in plants. This classification provides a simplified yet informative overview of the plant emission profile in relation to element content and environmental conditions.

Due to the non-homogeneous sampling design, which responded to the needs of the project in which it was framed, different data matrices were constructed for the chemometric approach based on several analytical objectives. For clarity and analytical consistency, this study focused only on two distinct datasets: (i) a total matrix of elements (36 × 22) consisting of elemental mass fraction data from leaf samples collected during Sampling 1, 2, and 3, and (ii) a combined matrix (12 × 29) containing both element and BVOC data from the September 2022 campaign. The latter was specifically developed to illustrate the application of chemometric techniques to a dataset that integrates information from two distinct analytical platforms. This combined dataset represents a typical case of data fusion in which complementary chemical information from different sources is integrated into a unified multivariate framework to enhance interpretability and analytical power. In general, data fusion approaches can be categorized into three main levels: (i) low-level data fusion, which involves the direct concatenation of raw or preprocessed data matrices from different sources; (ii) mid-level data fusion, where feature extraction is performed independently for each dataset prior to fusion, reducing dimensionality and emphasizing relevant information; and (iii) high-level data fusion, which combines the outputs of separate models or decisions derived from each data block [37,38]. In this study, a low-level data fusion strategy was adopted in which the standardized element mass fraction data and BVOC profiles were merged into a single matrix. This integrated dataset was then subjected to multivariate analysis to explore potential relationships between leaf elemental composition and volatile emission patterns and to assess whether the combined information could provide deeper insight into environmental monitoring. All the statistical analyses were performed using Matlab^®^ (The MathWorks, Natick, MA, USA) version R2024b and PLS_Toolbox 9.5 (2024) [39,40].

### 3.2. Multivariate Methods

#### 3.2.1. Data Preprocessing

As mentioned above, prior to applying chemometric methods and for effective data visualization, each data matrix was subjected to the appropriate preprocessing. This step is essential in the multivariate analysis of this type of data because it corrects differences in scale, reduces the influence of skewed distributions, and improves model interpretability and performance [41]. In this study, two main preprocessing strategies were considered. The first involved logarithmic, followed by mean-centering. The second method consisted of standard autoscaling, where the data were first mean-centered and then scaled to unit variance. Logarithmic transformation is particularly effective for handling right-skewed data and compressing large differences in magnitude, which are common in environmental datasets such as elemental mass fractions. The transformation was carried out using Equation (1), depending on the logarithmic base:

(1) $$x_{ij}^{\prime }={log}_{10}(x_{ij})$$

where $x_{ij}$ is the original value for variable *j* in sample *i*, and $x_{ij}^{\prime }$ is the transformed value. After transformation, as described above, the data were mean-centered. Mean-centering was performed using Equation (2):

(2) $$x_{ij}^{\prime }=x_{ij}-\bar{x_{j}}$$

where $\bar{x_{j}}$ is the mean of variable *j* across all samples. Alternatively, autoscaling was used when variables with different units or variances needed to be weighted equally in the model. Autoscaling involves mean-centering, followed by division by the standard deviation (Equation (3)):

(3) $$x_{ij}^{\prime }=\frac{x_{ij}-\bar{x_{j}}}{s_{j}}$$

where $s_{j}$ is the standard deviation of variable *j*.

In this work, the preprocessing strategies described above were applied and evaluated in the context of exploratory analysis. For PCA and PLS-DA, autoscaling was selected as the preferred pre-treatment method because it ensured equal variance contribution. In contrast, for PARAFAC modelling, all three preprocessing approaches (logarithmic transformation followed by mean-centering, using both log_10_ and natural log, and autoscaling) were tested. Exploratory results of notable relevance were obtained when log-transformed data were used, indicating that logarithmic preprocessing improved the interpretability and structure captured by the PARAFAC model.

#### 3.2.2. Principal Component Analysis

Principal Component Analysis (PCA) is a widely used exploratory technique that transforms a set of correlated variables into a new set of uncorrelated variables, known as principal components [20,30]. The goal was to reduce data dimensionality while preserving the maximum possible variance. This was achieved by changing the original coordinate system so that the first principal component (PC1) captured the direction of the greatest variance in the dataset. Subsequent components (PC2, PC3, …) are orthogonal to the previous ones and capture the remaining variance in a decreasing order of importance.

The decomposition of the original data matrix *X* in PCA can be expressed as Equation (4):

(4) $$X=TP^{T}+E$$

where *X* is the data matrix (samples × variables), *T* is the score matrix representing the coordinates of the samples in the principal component space, *P* is the loading matrix containing the weights (loadings) that define the principal components as linear combinations of the original variables, and *E* is the residual matrix representing the part of the data that is not explained by the retained components. By projecting the data into the space of the first few principal components, it is possible to visualize patterns such as clustering, outliers, and variable correlations. PCA is particularly useful for identifying major sources of variability, detecting redundant or irrelevant information, and preparing datasets for more advanced statistical modelling.

#### 3.2.3. Partial Least Squares-Discriminant Analysis

Partial Least Squares-Discriminant Analysis (PLS-DA) is a supervised classification technique derived from the PLS regression algorithm, which is specifically adapted for categorical response variables [19]. While PCA seeks directions that maximize the variance in the predictors without considering class membership, PLS-DA incorporates group information by projecting the data onto latent variables (LVs) that also maximize the covariance between the predictors (*X*) and response vector (*Y*). In particular, in PLS-DA, the class membership vector is transformed into a binary dummy matrix *Y* with n rows (samples) and G columns (classes). Classification is usually based on assigning each sample to a class from the predicted values for the response and the estimated probability of class membership. This makes it particularly effective in revealing patterns in complex datasets, where group structures may be obscured by high within-group variability. Mathematically (Equations (5) and (6)), PLS-DA models the data as follows:

(5) $$X=TP^{T}+E$$

(6) $$Y=UQ^{T}+F$$

*T* and *U* are the score matrices for *X* and *Y*, respectively. These represent projections of the original data onto the latent variable space. *P* and *Q* are the loading matrices, containing the weights that define the contribution of each original variable to the latent components. *E* and *F* are the residual matrices that capture the portion of the variance in *X* and *Y* that is not explained by the model.

In this study, PLS-DA was applied to classify samples according to spatial (leaf section: inner, intermediate, and outer) and temporal (sampling period) criteria similar to a supervised version of PCA [42]. Due to its supervised nature, PLS-DA is particularly useful in environmental monitoring, where large variability and overlapping groups may obscure meaningful patterns. By incorporating prior knowledge about group membership, PLS-DA enhances both interpretability and discriminatory power, making it a valuable complement to exploratory tools such as PCA.

#### 3.2.4. PARAllel FACtor Analysis

PARAllel FACtor (PARAFAC) analysis is a multi-way extension of Principal Component Analysis (PCA) capable of decomposing three-way data arrays into a sum of trilinear components, whereas Tucker3 is another such analysis [23]. In this study, PARAFAC was applied to a three-dimensional dataset (samples × elements × (places × time)). The model was constructed using the Alternating Least Squares (ALS) algorithm. Mathematically, the PARAFAC decomposition of a three-way array *X* is described by Equation (7):

(7) $$x_{ijk}=\sum_{f=1}^{F}a_{if}b_{jf}c_{kf}+e_{ijk}$$

where $x_{ijk}$ is the element of the data array at position (*i*, *j*, *k*); *F* is the number of factors; and $a_{if}b_{jf}c_{kf}$ are the loading coefficients for the three modes (e.g., samples, variables, and conditions). $e_{ijk}$ represents the residual error.

In environmental studies, PARAFAC allows for the simultaneous analysis of chemical, spatial, and temporal information without reducing the dataset to two dimensions, which may result in information loss. If the environmental data can be sorted into a dataset with more than two dimensions, they should be analyzed using a multi-way technique that considers this feature so that all possible internal relationships can be revealed. This makes it possible to better understand how pollutants act over time and at different environmental sampling sites, helping to uncover hidden patterns that simpler methods might overlook.

## 4. Conclusions

This study demonstrated that integrating chemometric techniques, ranging from classical unsupervised exploratory tools, such as PCA, to more advanced supervised (PLS-DA) and multi-way (PARAFAC) models, can significantly improve the interpretation of complex environmental monitoring data. When applied to real datasets from Mediterranean *Posidonia oceanica* and associated environmental matrices, these methods revealed spatial and temporal trends in element accumulation and volatile emissions that would have remained hidden using conventional univariate approaches. PCA enabled an initial unsupervised exploration of variability patterns, whereas PLS-DA improved group separation by incorporating prior class information. PARAFAC provided an effective means of modelling the three-way data structure, successfully distinguishing between baseline levels and meaningful compositional variations across space and time. Furthermore, the implementation of low-level data fusion, which combines trace element and BVOC information, proved valuable for enhancing analytical depth and revealing hidden patterns. This multi-level chemometric framework offers a reliable strategy for analyzing environmental datasets. Such approaches are especially useful in long-term monitoring programs, where large and heterogeneous datasets require scalable and interpretable solutions. By uncovering latent patterns and improving contribution clarity, these tools help make more informed environmental assessments and detect ecological stressors and pollution events more effectively.

## Figures and Tables

**Table 1:** Eigenvalues of the covariance matrix and percentage of variance explained by each principal component.

Component	Eigenvalue	% Variance
PC1	6.59	29.96
PC2	3.75	17.03
PC3	3.53	16.06
PC4	2.23	10.15
PC5	1.37	6.25
PC6	1.02	4.64
PC7	0.90	4.08

## Data Availability

The original contributions presented in this study are included in the article. Further inquiries can be directed to the corresponding authors.
